# Transient aphonia following spinal anesthesia during emergency cesarean section: Case report and review of literature

**DOI:** 10.1002/ccr3.7979

**Published:** 2023-10-06

**Authors:** Gauri Adhikari, Suson Ghimire, Gopal Adhikari, Krishnaraj Aryal, Narayan Kandel

**Affiliations:** ^1^ Department of Anaesthesia Nepalese Army Institute of Health Sciences‐ College of Medicine Kathmandu Nepal; ^2^ Department of Anaesthesia Patan Academy of Health Sciences (PAHS) Patan Nepal; ^3^ Department of Medicine Tribhuvan University Institute of Medicine Kathmandu Nepal

**Keywords:** intrathecal bupivacaine, spinal anesthesia, subdural block, transient aphonia, transient ischemic attack

## Abstract

Subarachnoid block with local anesthetic agents is a well‐established anesthesia technique among pregnant females for labor analgesia and cesarian delivery. Although it is considered a reliable and safe technique for both mothers and fetuses, unexpected high or low levels of block may occur due to accidental injection of these agents into different meningeal spaces other than intended. Hypotension, bradycardia, headache, and failed anesthesia are common complications of spinal anesthesia. Though rare, neurological complications like aphonia, dysphagia, and tingling sensation have also been reported. The article reports a case of a 22‐year‐old primigravida who sustained transient aphonia following intrathecal administration of bupivacaine for an emergency cesarian section for meconium‐stained liquor with fetal distress. There were no other neurological manifestations or features suggestive of high spinal block. The sensory level of the block was fixed to T6 with hemodynamic stability throughout aphasia with an episode of hypotension preceding aphonia. Aphonia commenced 9 min after the spinal anesthesia continued for a total duration of 15 min. Neurological examination, relevant investigations, and consultations were done to make a diagnosis. Thus, it is important to be aware of the possible neurological complications associated with spinal anesthesia.

## 1 INTRODUCTION

Spinal anesthesia has been in use for a long time in patients undergoing cesarean sections. The advantages of rapid administration, fast onset of action, and reduced risk of systemic toxicity and aspiration have made it the anesthesia of choice. However, it is not devoid of flaws, and complications like hypotension, bradycardia, headache, and failed anesthesia are commonly associated. Neurological complication, in contrast, is rare.^1^, ^2^

Aphonia is one of the rare neurological complications of spinal anesthesia. In the general population, it is commonly seen among females of age group 10 to 35 years of age with increased risk in women with poor educational background and low socioeconomic status.^3^, ^4^ Various etiologies and mechanisms have been proposed to define the cause of neurological complication; however, clear mechanism of many of them have not been identified yet.

We present the case of aphonia in a 22‐year‐old primigravida following the administration of bupivacaine during spinal anesthesia for an emergency cesarian section. The report has included the most probable cause of the condition with the sequential explanation of the differential to conclude the cause of aphonia in our case with the help of a review of relevant literature.

## 2 CASE REPORT

Twenty‐two‐year‐old primigravida was scheduled to undergo emergency cesarean section for delivery with the indication of meconium‐stained liquor with fetal distress. She weighed 64 kg and stood 166 cm. She had no history of psychiatric illnesses, seizures, other medical diseases, or drug abuse. Her antenatal period was unremarkable. On examination, her vital signs were normal, and the physical examination did not reveal any significant findings. The pre‐operative investigations, including electrocardiography (ECG) and routine blood investigation, which included complete blood count, liver function, kidney function, and coagulation profile, were within the normal range. At the labor ward, intravenous injections of Ranitidine 50 mg, Metoclopramide 10 mg, and injection Ondansetron 4 mg were given while the patient was being prepared. Upon arrival in the operating room, standard monitoring was initiated. Her blood pressure (BP) was 130/80 mm Hg, her pulse rate was 108 beats per minute (bpm), and her respiratory rate (RR) was 16/min. Ringer lactate (1000 mL) was infused intravenously before administration of the block. Under aseptic precautions, a subarachnoid block was given successfully with 25G Quincke's needle in a sitting position with a midline approach at L3/4 interspace in the first attempt without any trauma elicited. 2.2 mL (11 mg) of 0.5% hyperbaric bupivacaine was injected after confirming the free flow of cerebrospinal fluid (CSF). The patient was first kept supine with an immediate tilt to the left lateral position by placing a cloth wedge below the right hip. After spinal anesthesia, her BP was 134/88 mm Hg, PR of 112 bpm, RR of 18/min, and oxygen saturation (SpO_2_) of 99%. The level of sensory block to cotton swabs was T5.

However, 3 min after the subarachnoid block, she was found to be hypotensive with a BP of 70/36 mm Hg, and her heart rate was increased to 158 beats/min. She was managed with a rapid infusion of 250 mL of RL and an injection of Mephentermine (12 mg in two divided doses), the only available drug in the setting. After management, her BP rose to 108/66 mm Hg, and the rest of her vitals were also normal. During the hypotensive period, she had no new complaints of dizziness or nausea. Surgery was started, and a 2.6 kg baby boy with an APGAR score of 8/10 at 1 min and 9/10 at 5 mins was delivered.

After 3 min of delivery, the patient was found to be uttering some sound. She had her facial expressions intact, was alert and trying to communicate, and then, within a minute, she developed aphonia. She seemed anxious and moved her upper body parts and head from side to side. Her vitals were stable; thus, she was reassured, and oxygen was administered with a simple oxygen mask at the rate of 5 L/min, and the surgeons were allowed to proceed with surgery. There were no signs of motor blockade in the upper extremities, and the patient could move her upper limbs completely with a firm hand grip. Although anxious, she was awake, alert, and could follow commands (asked to protrude tongue). The level of sensory block was reassessed and found to be fixed at T6. The aphonia gradually resolved after 15 min, and she could vocalize. The rest of the surgery was uneventful.

Postoperatively, the patient was monitored in the recovery room until the motor blockade of the lower extremities was reversed. Neurological examination was normal. ECG was done, which was normal. The neurological examination did not reveal any abnormality. Ultrasound doppler of the lower leg veins, carotid artery doppler, transthoracic echocardiography, and electroencephalography (EEG) were done. There was no abnormality detected in any of the tests. The patient was discharged with no sequelae of any kind after 3 days.

## 3 DISCUSSION

Aphonia is a complete inability to produce the sound that occurs after bilateral palsy in the abduction of vocal folds. The ipsilateral recurrent laryngeal nerve (RLN) is responsible for the motor innervation of muscle abduction of the vocal folds. Unilateral RLN injury paralyzes the ipsilateral vocal fold, and the clinical symptoms of dysphonia or hoarseness appear. If the RLN paralysis or injury is bilateral, then aphonia develops due to paralysis of both vocal folds.^5^, ^6^ RLN is the branch of the vagus nerve, and the incidence of such a condition due to vagus nerve paralysis is reported to be 1:200 to 1:1200.^2^

The incidence or prevalence of aphonia in patients undergoing surgery with spinal anesthesia is not well known. However, it is a common complication of head and neck surgery, like thyroidectomy. Aphonia during spinal anesthesia can be triggered by a wide range of conditions like transient ischemic attack (TIA), absent seizure, subdural block, high spinal block, intrathecal lipophilic opioid injection, and conversion disorder along with cerebrovascular complications of pregnancy due to preeclampsia or eclampsia.^4^, ^7^, ^8^, ^9^

American Stroke Association defines TIA as “a transient episode of neurologic dysfunction caused by focal brain, spinal cord or retinal ischemia, without acute infarction.” It can occur due to hypotension, hypoxia, or embolism that compromises cerebral or vertebrobasilar circulation. TIA signs are temporary vision loss, aphasia, hemiparesis, and paresthesia, usually occurring on one side of the body.^4^, ^7^, ^8^ Hypotension is a known complication of spinal anesthesia, and its incidence in parturients can be as high as 80%.^1^ In this case, the patient had an episode of hypotension, which could have decreased blood supply to the brain, leading to TIA. Thromboembolism or amniotic fluid embolism as a cause of TIA is a rare event during pregnancy with variable presentation.^8^ Other than aphonia, our patient did not have any other features of TIA, and ultrasound doppler of the lower leg veins for deep vein thrombosis and carotid artery Doppler for arterial embolus were normal. Additionally, ECG and transthoracic echocardiography were normal, which ruled out atrial fibrillation or patent foramen ovale, which are possible sources of embolus. Furthermore, during aphonia, the patient could control her facial movements and move her lips, tongue, and eyes with intact vision. She did not manifest weakness in her upper limb, assessed with a firm hand grip. The lower limb was under the effect of anesthesia and hence could not be assessed. MRI could not be done due to unavailability. Therefore, based on the results, the likelihood of transient ischemic attack (TIA) being this patient's underlying cause of aphonia is minimal. However, it could not be entirely ruled out as there is a possibility of TIA due to the presence of hypotension, which could potentially contribute to the condition.

The patient did not have a history suggestive of similar illness in the past, anytime in the child or adulthood. Hypotension leading to hypoxia in the brain can be the possible cause of absence seizure, but the patient was alert, was trying to communicate, and was hemodynamically stable. Additionally, her EEG post‐surgery did not show any epileptiform activities, thus ruling the absence of seizure. Since our patient did not have a history and presentation suggestive of hypertensive disorder of pregnancy, preeclampsia, or eclampsia as the possible cause of cerebrovascular complication leading to aphonia, these conditions were ruled out.

Subdural space extends into the cranial cavity throughout the distribution of the meninges, covering all neural structures. The onset of the block is between that of a subarachnoid and epidural block.^10^ Subdural injection of the drug has varied presentations such as excessive sensory blockade with sparing of sympathetic functions, failed spinal anesthesia, significant motor weakness of upper extremities, significant hypotension, and delayed or faster than usual onset of block.^1^, ^10^ Sensory block is usually high and disproportionate to the volume of drug injected, as the limited capacity of the space results in extensive spread. There is usually sparing of, or minimal effect on, sympathetic and motor functions due to relative sparing of the ventral nerve roots. Since the subdural block extends cranially, a local anesthetic block of the brainstem is possible, causing loss of consciousness, aphonia, and apnea.^10^ Transient aphasia, in our case, could have been due to the effect of bupivacaine on the brain stem. Studies have reported unintended subdural block with the use of reusable long beveled needles, as in our case, and with multiple attempts while administering the block, which was not present in our case.^10^, ^11^ However, the probability of the needle tip lying partly in subarachnoid and subdural space has to be considered. A definitive diagnosis of the condition requires computed tomography, which was not available in our setting; we can assume from the clinical scenario that subdural block can also be one of the causes of aphonia in our case.

Lipophilic opioids such as fentanyl, often used as an adjuvant to local anesthesia for intrathecal administration, have been shown to be associated with facial numbness, dysphasia, and aphonia.^1^ The most likely mechanism is due to the rostral spread of the drug to the speech area or cranial nerves, and the transient nature of the symptom is due to the rapid clearance of the drug from the CSF.^12^ Studies have shown cases where aphonia alone or accompanied by neurological symptoms have occurred after spinal anesthesia in pregnant females and other surgeries.^1^, ^7^, ^11^, ^12^ However, no opioid was used in this case, excluding it as a cause of aphonia.

A wider pelvis during pregnancy makes the vertebral column inclined toward the head end in the lateral position, which may increase the cephalad spread of the drug, leading to a high spinal block.^1^ Though sudden hypotension occurred in our patient, other features of high sympathetic blockade, such as respiratory distress, bradycardia, and pupillary dilatation, were absent.^1^, ^10^ Thus, the high spinal block was ruled out in our patient as she was completely awake, hemodynamically stable, maintaining normal saturation, and tachycardic throughout the period, with the sensory level of block fixed at T6.

Most cases of conversion disorder have been linked to general anesthesia.^3^, ^4^ The cases are not only limited to general anesthesia but also present in regional anesthesia, as mentioned in a literature review by Kwok. In many of these cases, patients had no history of psychological or psychiatric disorders previously, and they were diagnosed as a case of conversion disorder after ruling out the possible cause of aphonia.^4^ Interestingly, the presentation of these mentioned cases had a variety of presentations, and the duration of recovery was also varied. Though the risk factors, age between 10 and 35 years of age and female gender are present in our case, her lack of similar history in the past, good educational background with school level education, and good socioeconomic status make this diagnosis unlikely.^3^, ^4^, ^7^

Thus, in our case, the cause of aphonia was speculated to be either TIA or subdural block.

## 4 CONCLUSION

In conclusion, neurological events such as aphonia can occur in any surgery involving spinal anesthesia. Various etiologies from TIA to conversion disorders and the onset, duration, and time to recovery have been reported from minutes to days. So, it is crucial to exclude all the possible causes to come to a definitive diagnosis, considering the history and presentation of the patient. Additionally, it is to be remembered that accidental subdural injection during subarachnoid block is possible. Furthermore, the possibility of multiple conditions must also be considered during the management and follow‐up of the patient.

## AUTHOR CONTRIBUTIONS

**Gauri Adhikari:** Writing – original draft; writing – review and editing. **Suson Ghimire:** Conceptualization; supervision; validation. **Gopal Adhikari:** Project administration; writing – original draft. **Krishnaraj Aryal:** Resources; writing – review and editing. **Narayan Kandel:** Writing – review and editing.

## CONFLICT OF INTEREST STATEMENT

The authors have no conflict of interest.

## ETHICS STATEMENT

Consent was taken from the patient herself for the write‐up and publication of this paper.

## CONSENT

Written informed consent was obtained from the patient to publish this report in accordance with the journal's patient consent policy.

## Data Availability

Data sharing is not applicable ‐ no new data is generated, or the article describes entirely theoretical research.
